# Consistency and changes in eye movements during static, dynamic, and audio-visual emotion processing

**DOI:** 10.1177/03010066261439924

**Published:** 2026-04-29

**Authors:** Holly Cooper, Ben J. Jennings, Veena Kumari, Caroline Di Bernardi Luft, Rachel J. Bennetts

**Affiliations:** 1 Centre for Human Brain Health, 3890 School of Psychology, University of Birmingham, Birmingham, UK; 2 College of Health and Society, 3890 Buckinghamshire New University, Buckinghamshire, UK; 3 Department of Psychology, College of Health, 3890 Medicine and Life Sciences, Brunel University of London, London, UK

**Keywords:** emotion recognition, facial expressions, audio-visual expressions, individual differences, eye movement patterns

## Abstract

Tracking eye movements as individuals view emotional facial expressions can offer insight into the mechanisms underlying emotion recognition. Few studies compare eye gaze patterns across static and dynamic expressions, and even fewer use ecologically valid audio-visual stimuli. Research is also limited on individual differences in emotion processing and how these relate to recognition performance. In this study, we address this gap by examining the eye movement patterns of adult participants (*n* = 73) during emotion recognition of individuals in static photos, dynamic videos, and audio-visual videos. Using pre-defined region-of-interest analyses and Hidden Markov Models, we explored whether individuals focused on different areas of the face across conditions, whether individual eye movement patterns were consistent across conditions, and whether certain patterns were associated with better performance. The findings showed that a) individuals focused more on the eye region when recognising static expressions compared to moving expressions; b) individuals’ eye movement patterns during emotion recognition were highly consistent, albeit most likely to change between static and moving conditions; c) there was not one particular pattern associated with improved emotion recognition accuracy. Ultimately, we report that the emotion recognition of static emotional expressions differs from moving expressions, suggesting recognition is influenced by movement, highlighting important considerations for future research to include more realistic stimuli to be better able to generalise to real-world social interactions.

## Introduction

Recognising emotional displays and adapting our behaviour accordingly is a fundamental component of our social interactions (Marinetti et al., 2010). Accurate emotion recognition is a key element in this process, and examining eye movements as people view emotional faces can help us understand the processes underpinning accurate emotion recognition. To date, most research exploring eye movement patterns in emotion recognition has used static (a still, single image) facial expressions (Murphy & Isaacowitz, 2010; Eisenbarth & Alpers, 2011; Wells et al., 2016). However, static stimuli lack some of the information that is present in real-world interactions – for example, information about the development and temporal characteristics of the expression, and vocal cues of emotion – which presents issues for ecological validity. Thus, more generalisable stimuli, such as moving expressions and stimuli which include auditory information, are ideal for understanding how emotions are processed in real-life interactions (Alves, 2013). In this paper, we examine patterns of eye movements during emotion recognition using static images, dynamic videos, and audio-visual videos (with visual and auditory cues) displaying different emotional expressions (EEs). To explore eye movements to information-rich facial features (e.g., eyes and mouth; Eisenbarth & Alpers, 2011, Calvo et al., 2014), as well as accounting for temporal information and individual differences, we use a combination of pre-defined region-of-interest (ROI) analyses and Hidden Markov Models (HMMs) to examine 1) patterns of eye movements in each condition (static, dynamic, and audio-visual); 2) the stability of eye movements across these different conditions; and 3) how these eye movements are associated with accurate emotion recognition.

It is well-established that movement cues facilitate face processing. For example, in the face identification literature, there is a ‘movement advantage’: participants are better able to identify and match moving faces compared to static faces (O’Toole et al., 2002; Bennetts et al., 2013). Some studies suggest that there is also a ‘dynamic advantage’ in EE recognition (Fiorentini & Viviani, 2011; Jiang et al., 2014; Richoz et al., 2024), where dynamic EEs are more accurately recognised than static EEs. In support of this ‘dynamic advantage’, studies comparing static and dynamic emotion recognition have reported that participants are better at recognising dynamic EEs compared to static EEs, and conclude that the presence of movement aids emotion recognition (Ambadar et al., 2005; Bould & Morris, 2008). This effect can be observed across the life span (from age 5 to 96; Richoz et al., 2018).

The dynamic advantage is particularly evident in situations where observers struggle with emotion recognition (see Krumhuber et al., 2023, for a comprehensive review). The inclusion of movement can attenuate emotion recognition deficits typically seen in older adults (Holland et al., 2019), clinical samples such as individuals with schizophrenia and depression (Garrido-Vasquez et al., 2011), as well as individuals with neurological disorders such as autism spectrum disorder (Gepner et al., 2001). Motion can also help when the stimuli are ambiguous, such as low-quality or share similar visual properties with another expression (e.g., fear and surprise; Krumhuber et al., 2023).

There is also evidence refuting this ‘dynamic advantage’ and reporting similar accuracy across static and dynamic EEs (Fiorentini & Viviani, 2011). This seems to be particularly evident with younger samples; 3 to 5 year olds (Nelson & Russell, 2011) and 5 to 10 year olds (Widen & Russell, 2015). Thus, the behavioural literature is conflicting. However, reviews by Alves (2013) and Krumhuber et al. (2023) compared the recognition of static and dynamic facial EEs using various techniques beyond behavioural measures. They both reported that dynamic EEs have better accuracy than static EEs, and suggested stronger involvement of certain brain areas, and a larger network, when processing dynamic EEs compared to static EEs.

The idea that static and dynamic EEs are processed differently is also supported by research on eye movements and emotion recognition. Prunty et al. (2021) provide evidence for this difference emerging early in life. Infant's eye movements were recorded when viewing dynamic EEs (videos of actors expressing an emotion) and static EEs (stills taken at peak expression), and differences between conditions were found. Overall infants spent more time looking at dynamic EEs than static EEs, and they focused more on the lower half of the face for dynamic EEs compared to static EEs. Blais et al. (2017) compared eye movements for static and dynamic facial EEs in adults. The dynamic videos included actors displaying a facial EE and the static images were the apex of the EE demonstrated in the video. Overall, participants had better accuracy for dynamic compared to static EEs. Regarding eye gaze, for dynamic faces, gaze stayed in the centre of the face and showed fewer fixations on the eye and mouth regions compared to static faces, with which gaze quickly spread outward (Blais et al., 2017).

There are few studies which directly compare individuals’ eye movement patterns between static and moving EEs, and even fewer which incorporate audio-visual EEs (Zheng & Hsiao, 2023). Thus, it is unclear how audio may interact with emotion recognition abilities and eye gaze patterns. As such, the first aim of the study is to examine, on a group-based level, whether movement and audio cues affect the proportion of time individuals spend examining different areas of the face.

### Individual Differences in Eye Movements

Although important to explore eye movements at group-level (e.g., aggregating across participants), this does not account for inter-individual variation in visual information processing patterns. Growing evidence suggests that individual differences in visual processing matter: atypical gaze patterns are associated with meaningful differences between individuals, including traits linked to autism, social anxiety, phobias, and schizotypy traits (Kleberg et al., 2017; Ni et al., 2023; Thomas et al., 2024).

Recently, a number of studies have started to explore individual differences in eye movements – for example, where different people might fixate when they first look at a face (Peterson & Eckstein, 2013); different processing patterns associated with face processing (e.g., more ‘holistic’ vs more ‘analytic’ patterns of eye movements; Chuk et al., 2014); how consistent these patterns are across tasks and conditions (Meaux & Vuilleumier, 2016; Rodger et al., 2023); and how they relate to performance on a task (Richler et al., 2011; Yüvrük et al., 2024; Paparelli et al., 2024). Some of these studies (Chuk et al., 2014; Paparelli et al., 2024) go beyond comparing group-averaged fixations between static and dynamic EE stimuli and have started to explore how individual differences might be associated with performance.

For example, Paparelli et al. (2024) employed HMMs to explore whether specific eye movement patterns were related to better performance in an emotion recognition task. They presented only the face region, in black and white, displaying each of these emotions: anger, disgust, fear, happiness, sadness, and surprise. The dynamic stimuli started neutral and progressed to an EE over the course of 30 frames, while the static stimuli showed the last frame (the apex of the EE). Fixation patterns across the EEs were analysed using HMMs (Chuk et al., 2014), a probabilistic approach which can be used to explore latent states in gaze sequences. This revealed three distinct groups and scanning patterns: group 1′s fixation started around the mouth, left eye, and corner of right eye, group 2′s started towards the mouth and partly the left eye and then more central around the nasion to mouth, and group 3′s started towards the left eye and then the midline of the face or stayed around the eyes. There were minimal differences in performance across scanning patterns, suggesting that successful emotion recognition did not depend on a specific eye movement pattern. Furthermore, when categorising participants’ patterns, they found that most participants developed a single effective scanning pattern which they employed with both static and dynamic emotion recognition, meaning individuals’ processing patterns tended to fall into the same group of scanning patterns, regardless of how the stimuli were presented. This is somewhat surprising as previous research exploring dynamic and static EEs reported that fixations for static stimuli were more widespread, and dynamic were more central focused (Blais et al., 2017). Although, Blais et al. (2017) reported averaged group-level effects whereas Paparelli et al.'s (2024) analyses explored more subtle within-person effects. However, Paparelli et al. (2024) suggested that methodological factors could explain this difference in findings, as previous studies used a shorter presentation time (e.g., 500 ms in Blais et al., 2017), while theirs was 1 second.

Paparelli et al.'s (2024) study provides some insight into whether individuals’ processing patterns are consistent when viewing static or moving EEs, and the relationship between processing patterns and performance. However, the stimuli were still relatively limited in their ecological validity as they were presented in black and white and cropped at the hairline to only show internal facial features. Further, these stimuli examine EE recognition in an isolated and unimodal context, whereas real-world EEs are often conveyed during an interaction (e.g., while a person is speaking), and communicated via multiple channels (e.g., visual and auditory).

To the best of our knowledge, there is only one study which examines individual differences in eye movement patterns using more realistic stimuli. Zheng and Hsiao (2023)^1^ explored whether the processing of vocal EEs interfered with the eye movements for facial EEs. Participants viewed EEs in three conditions: (1) audio-only (vocal EE with a static neutral face displayed), (2) video-only (facial EE without vocal content), and (3) audio-visual (both facial and vocal EE present), and their data were analysed using HMMs. They reported that accuracy was highest in the audio-visual condition compared to audio- and video-only conditions, but eye movement patterns did not significantly differ between conditions.

Zheng and Hsiao's (2023) study provides important information about how visual and vocal cues combine during the processing of EEs. However, the analyses were focused on examining the audio-visual advantage – as such, the study provides limited information about the role of movement and the dynamic advantage in facial EE recognition. The audio-only condition included a static neutral face paired with a vocal EE, which creates a bimodal EE (as both vocal and visual elements are included). Previous research has shown that the presence of information in one modality can influence the other, e.g., a facial EE can influence the recognition of a vocal EE and vice versa (Collignon et al., 2008; Cooper, 2023), similar to the McGurk effect (an audio-visual illusion where looking at facial movements influences what speech sounds people hear; McGurk & MacDonald, 1976). Therefore, it remains unclear whether individuals adopt different eye movement patterns when they are processing audio-visual expressions versus dynamic facial EE alone, how each of these conditions compares to static EE recognition, and how eye movement patterns are associated with accuracy in each of these conditions.

To truly understand how we recognise EEs in others, we need to move beyond artificial stimuli and embrace more realistic ones – especially dynamic and audio-visual EEs that better reflect typical social interactions. Such stimuli allow us to test whether people's eye movement patterns remain stable across formats (static, dynamic, and audio-visual), or whether these patterns shift depending on the richness of emotional cues. Thus, the current study seeks to bridge key gaps in our understanding by examining how eye movement patterns vary – or stay consistent – across different modes of EEs.

### The Current Study

The current study addresses three key questions: (1) do individuals focus on different areas of the face when recognising static, dynamic, and audio-visual EEs; (2) are individuals’ eye movement patterns consistent across static, dynamic, and audio-visual EEs; and (3) are specific processing patterns associated with better emotion recognition accuracy.

Any hypotheses concerning audio-visual expressions are cautious due to the lack of research in general, as well as a lack of appropriate comparison across static, dynamic, and audio-visual conditions (but see Zheng and Hsiao, 2023). However, for the first research question, it is hypothesised that static EEs will show a wider spread of eye movements than moving EEs (dynamic and audio-visual; Blais et al., 2017). Regarding the second and third research questions, we hypothesise that participants will favour the same processing pattern across both static and dynamic conditions (Paparelli et al., 2024), and no specific processing pattern will be associated with better performance (Paparelli et al., 2024).

## Methods

### Participants

The sample consisted of 73 participants (53 female; 20 male, M_age_ = 22 years, *SD* = 5.42). In the sample, 38 participants identified their ethnicity as Asian / Pacific Islander (52.1%), 20 identified as White (27.4%), 10 identified as Other (13.7%), 4 identified as Black (5.5%), and 1 identified as Native American or American Indian (1.4%). The sample size follows previous eye tracking and recognition work which has employed the same data-driven analyses (Bennetts et al., 2025; Paparelli et al., 2024; Zheng & Hsiao, 2023). Participants were recruited from the undergraduate psychology cohort at Brunel University London in exchange for 3 course credits or through opportunity sampling in exchange for a £7.50 voucher. The inclusion criteria were aged between 18 and 50 years, normal or corrected-to-normal vision, no significant hearing loss that would render daily tasks and conversations difficult, and fluent in English (due to a verbal IQ test – Wechsler Test of Adult Reading; Wechsler, 2001 – including unusually spelt English words, which was collected as part of a larger project, but not analysed here). As differences in eye movement processing patterns have been linked to cognitive decline (particularly visuo-spatial attention) in older adults, the maximum age was 50 years old (Chan et al., 2018). Ethical approval was granted by the Research Ethics Committee for the College of Health, Medicine, and Life Sciences at Brunel University London.

### Materials and Apparatus

**Figure 1.**
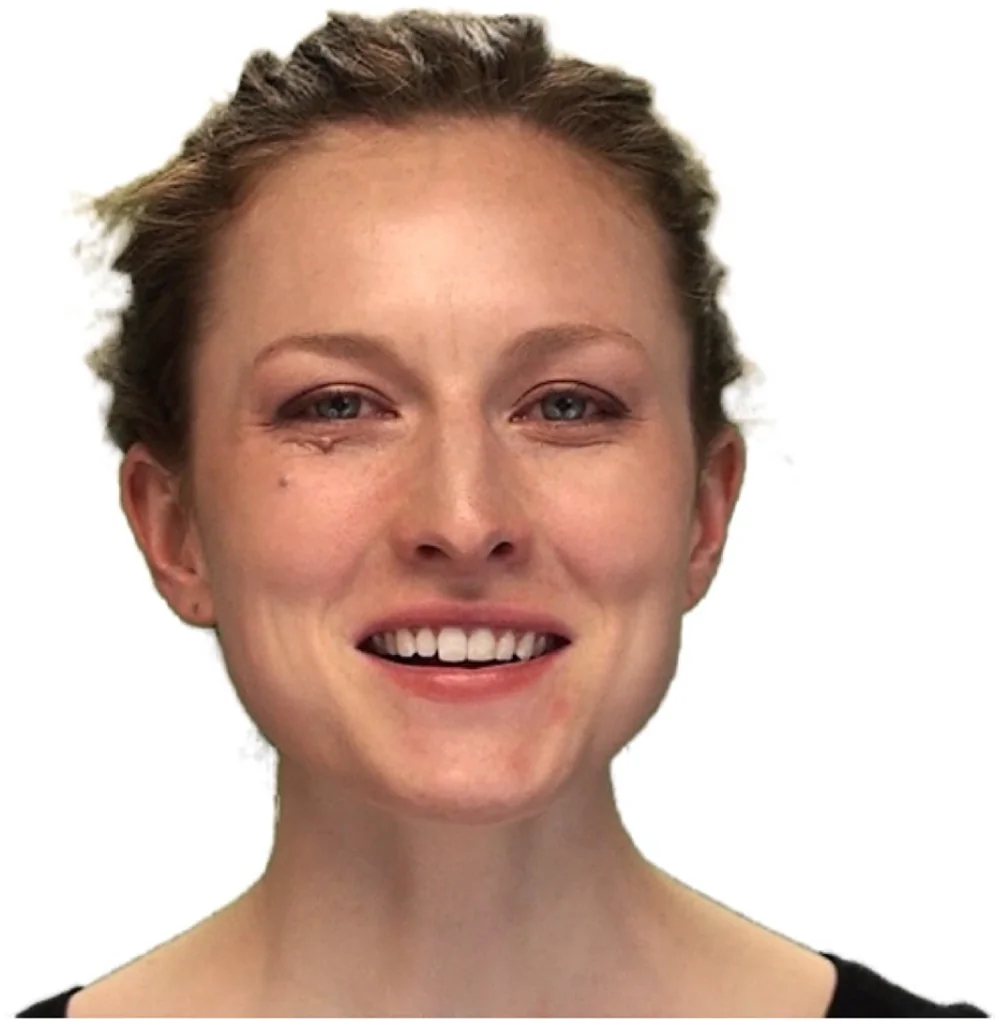
Static stimulus example showing actor 2′s portrayal of happiness.

The EE stimuli were selected from the Ryerson Audio-Visual Database of Emotional Speech and Song (RAVDESS; Livingstone & Russo, 2018). This database includes the basic six emotions (happy, sad, anger, fear, disgust, and surprise) and a neutral condition across three modalities (visual, audio, and audio-visual EEs), and with two intensities (normal and strong) for each of the six basic emotions. The authors have previously explored emotional intensity in relation to accuracy (Cooper et al., 2024), but as there is no theoretical justification for eye movements differing across intensity levels, it was not explored here. The stimuli used in the current study included static, dynamic, and audio-visual EEs. In all conditions, the stimuli showed an actor's face and the top of their shoulders with black t-shirts on a white background (Figure 1). For the audio-visual stimuli, the actors said the semantically neutral sentence of ‘dogs are sitting by the door’, the videos were approximately 3 to 4 seconds long. The dynamic stimuli were identical to the audio-visual stimuli, but without the audio. The dynamic facial EEs and the audio-visual EEs were taken exactly as they were from the RAVDESS. As this database did not include static EEs, we created them using the apex of the dynamic EEs, similar to previous studies (Blais et al., 2017; Paparelli et al., 2024). To keep timings consistent, the static EEs were also presented for 4 seconds. When presented on screen, the faces were approximately 10 cm wide and 13 cm tall. The horizontal visual angle was approximately 8 degrees, and the vertical visual angle was approximately 10 degrees.

A total of three identities (1 male, 2 females: actors 2, 7, 12) were used in the main task. Three different identities (2 males, 1 female: actors 8, 17, 23) were used for the practice trials. There was a total of 117 trials (13 stimuli (basic six emotions x 2 intensities, and neutral) x 3 actors x 3 modalities).

The experiment was programmed and displayed using SR Research Experiment Builder software (SR Research Ltd., Kanata, ON, Canada), running on a desktop computer using Windows 7 (Microsoft, Inc.). The stimuli were displayed in the centre of a 21-inch colour CRT monitor (Dell P1110) with the screen resolution set to 1,280 × 1,024 pixels at a vertical refresh rate of 100 Hz. Viewing distance was held constant at 75.5 cm with a chin rest. Participants wore headphones so the audio in the audio-visual EEs could be controlled (maximum volume on the speaker and computer volume at 25).

Eye movements were recorded using an EyeLink 1000 desk-mounted video-based eye-tracker, which recorded eye movements at 1,000 Hz (SR Research Ltd., Kanata, ON, Canada). A nine-point calibration procedure at the beginning of each block, and a drift correct (a dot in centre of screen) before each trial ensured participants’ eye movements were calibrated and centred at the beginning of every trial.

### Procedure

**Figure 2.**
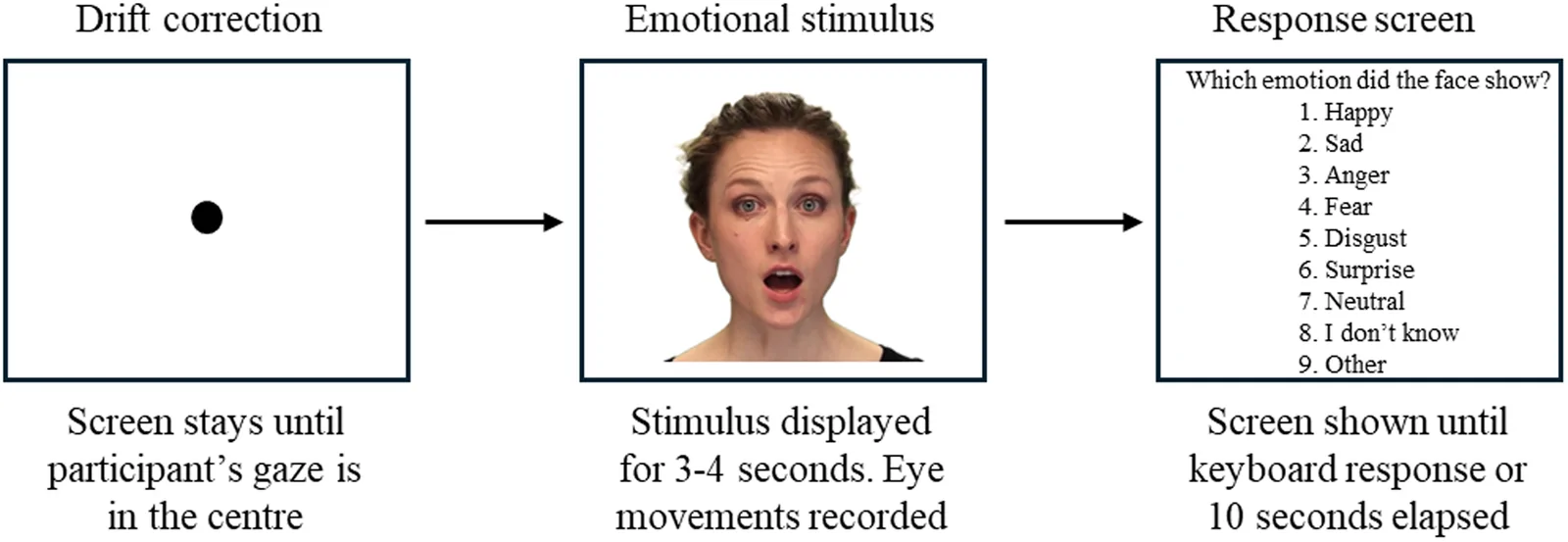
Experimental trial design including each screen and key details.

Each trial began with participants focusing on a dot in the centre of the screen. Once the experimenter initiated the trial, a single image or video appeared in the middle of the screen, lasting approximately 4 seconds. After this, the stimulus disappeared and was replaced by the response screen, showing response options of Happy, Sad, Anger, Fear, Disgust, Surprise, None/Neutral, I don’t know, and Other. Participants responded using keyboard numbers. The emotion labels were always presented in the same order and associated with the same numbers. Although this could have resulted in response bias, high accuracy rates suggest that participants were using the full range of responses. Once participants responded (or 10 seconds had elapsed), the experiment moved onto the next trial. If no option was selected within 10 seconds, no response was recorded and the trial was marked as incorrect. See Figure 2 for a pictorial explanation. Participants were not able to respond during stimulus presentation. Participants were asked to refrain from moving or talking during the trials, and to try to not look at keyboard before the response screen. Only eye movements from the period the emotion stimuli were being displayed (approximately 4 seconds per trial) were subsequently included in the analysis, to avoid tracking gaze to the keyboard when responding.

The three conditions (static, dynamic, audio-visual EEs) were blocked, and trials within each block were presented in a fixed randomised order. Each block lasted approximately 6 min. The blocks were not counterbalanced: the conditions were presented from least (static) to most cues (audio-visual) to avoid inflated performance. For example, if participants saw the audio-visual EEs first (which contain multiple cues to the emotion), it may have made it easier to identify the same emotion again when the clip was shown without the audio/movement information. Three practice trials were presented before initial calibration in each condition. The practice trials had the same procedure, response options, and screens as the main task.

### Data Analysis

**Figure 3.**
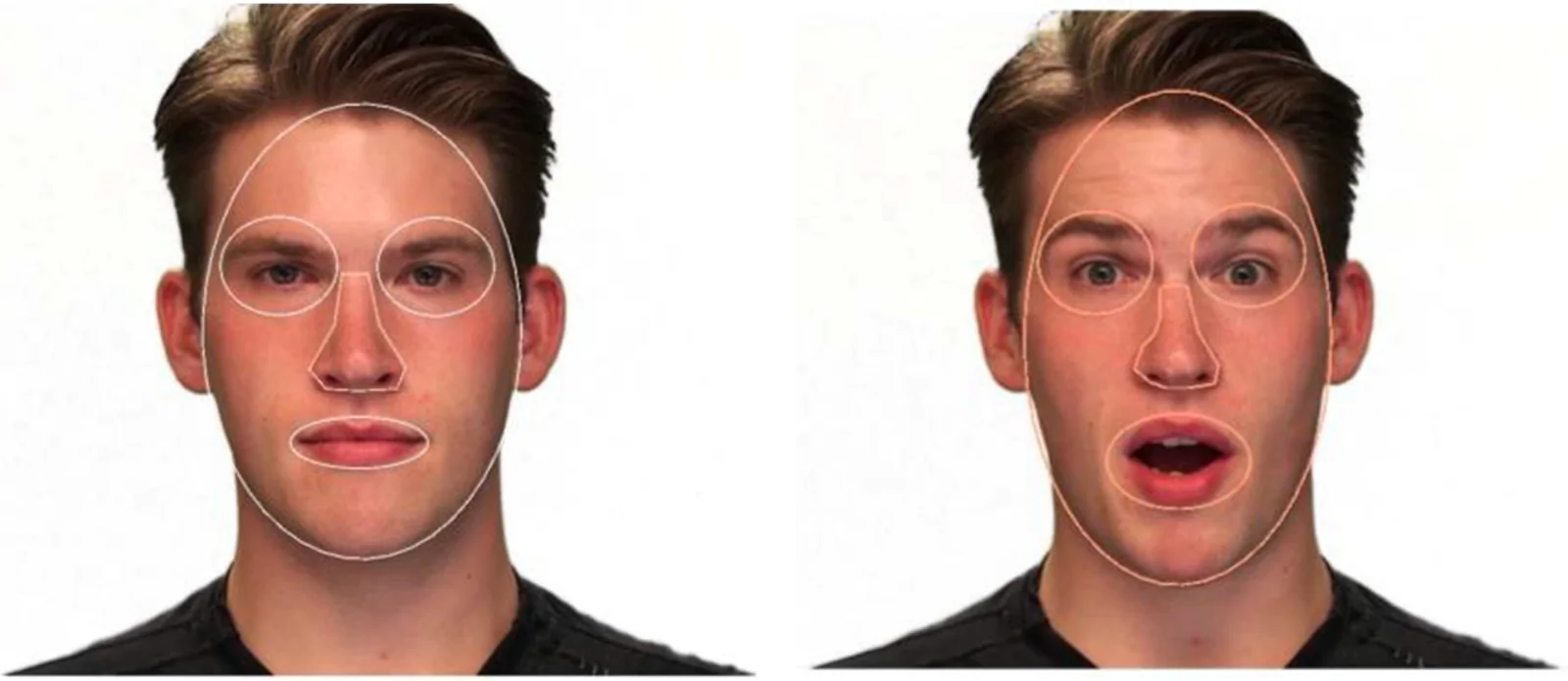
Examples of the pre-defined ROIs for actor 7′s expressions of neutral (left) and surprise (right). ROI: region-of-interest.

First, we carried out a two-way analysis of variance (ANOVA) to explore dwell time across stimulus presentation (static, dynamic, audio-visual) and pre-defined ROIs (eyes, nose, mouth, and non-feature regions), to examine where participants were focusing on the face when viewing EEs. The main analyses did not include emotion type as a factor as previous research found no significant differences between EEs (Blais et al., 2017). Our pre-defined ROIs were manually created for each of the stimuli: left eye, right eye, nose, mouth, and the non-feature regions (Figure 3). The non-feature regions included the facial areas excluding the eyes, nose, and mouth. These areas were consistent across conditions. The ROIs for the dynamic and audio-visual conditions were dynamic (e.g., they moved and changed as the head and features moved during the EE), so the ROIs were manually adjusted every *n*th frame in line with the amount of movement - EEs with less movement required fewer readjustments compared to EEs with more movement. The dynamic and audio-visual EEs shared the same manually created ROIs as the visual element of the conditions was identical.

Secondly, eye movements were analysed using the Eye Movement analysis with Hidden Markov Models (EMHMM; Chuk et al., 2014), to explore whether eye movement patterns were consistent across modalities. The EMHMM approach has been used to examine common patterns of eye movements, and how these relate to individual differences, in a variety of face processing tasks (e.g., Chan et al., 2018; Chuk et al., 2014; Chuk et al., 2017a; Chuk et al., 2017b; Paparelli et al., 2024; Zheng & Hsiao, 2023). Broadly, HMMs incorporate both spatial (fixation co-ordinates) and temporal (fixation order) information, modelling individuals’ eye movements as hidden ‘states’ (individual ROIs) and the transitions between them. The EMHMM process first derives a HMM for each datapoint (e.g., each participant, or in some cases data from one experimental condition per participant) using a variational Bayesian expectation-maximisation algorithm; subsequently, it clusters the individual HMMs into groups using a variational hierarchical expectation-maximisation algorithm, to discover common patterns of eye movements within the data (Chuk et al., 2017b). The EMHMM toolbox, containing the code to run HMMs on eye movements, was obtained from the Video, Image, and Sound Analysis Lab's website (http://visal.cs.cityu.edu.hk/research/emhmm/). The dataset had 219 data points (73 participants x 3 modalities). For each participant, the algorithm automatically selected the optimum number of ROIs for the individual HMMs from a pre-defined range from 1 to 3 (as used in previous papers, e.g., Chuk et al., 2014; Paparelli et al., 2024). Subsequently, the median number of ROIs in the individual HMMs was adopted for the group HMMs. Once the group HMMs had been derived, we calculated the log-likelihood of each individual HMM belonging to each group HMM. This acted as a measure of similarity or match between the individual's eye movement pattern and the group pattern. To determine if the clusters are truly different, we compared log-likelihood patterns for the groups (e.g., how similar on average group 1 is to the group 1 HMM; compared to how similar on average group 2 is to the group 1; and the same for the group 2 HMM) using t-tests. To determine the appropriate number of groups, we followed Paparelli et al.'s (2024) method of increasing the number of groups until the difference between log-likelihoods for each group was no longer significantly different. These analyses indicated that the data were best summarised by two groups of eye movement patterns.

Third, we carried out a two-way ANOVA to explore average emotion recognition accuracy (proportion correct) across eye movement pattern group (1 or 2) and stimulus presentation (static, dynamic, audio-visual). When calculating accuracy for the third research question, free-labelling in ‘Other’ and ‘I don’t know’ responses were classified as incorrect to ensure consistency with how the database labelled the emotions. Less than 1% of responses were ‘Other’ or ‘I don’t know’.

Assumption checks were conducted. Any violations and corrections are discussed before each analysis. Multiple comparisons were corrected using Tukey's test.

The static stimuli we created and the data files generated and analysed for the current study are available on Open Science Framework: (https://osf.io/vyg7p/). The moving stimuli are from Livingstone and Russo (2018), available at https://zenodo.org/records/1188976.

## Results

The results are split into three sections in line with the research questions. The descriptives are presented in Table 1.

**Table 1.** Descriptives for pre-defined ROI (eyes, nose, mouth, non-feature regions) and stimulus presentation (static, dynamic, audio-visual) displaying mean (standard deviation) of proportion of dwell time.

ROI	Static	Dynamic	Audio-Visual
Eyes	0.39 (0.19)	0.32 (0.24)	0.34 (0.24)
Nose	0.21 (0.13)	0.22 (0.18)	0.22 (0.18)
Mouth	0.14 (0.12)	0.16 (0.20)	0.14 (0.19)
Non-feature regions	0.25 (0.09)	0.28 (0.09)	0.28 (0.09)

ROI: region-of-interest.

**Research question 1: Do individuals focus on different areas of the face when recognising emotions from static, dynamic, and audio-visual EEs?**

A two-way ANOVA was employed to examine the proportion of dwell time to pre-defined ROI (eyes, nose, mouth, non-feature regions) and whether this differs depending on how the EEs were presented (static, dynamic, audio-visual). The Q-Q plot indicated slightly non-normal data and as such outliers were explored. Looking across conditions, the outliers followed a pattern: select participants showed higher dwell time to the mouth region across conditions. Thus, these ‘outliers’ more likely reflect individual differences (preference for the lower half of the face compared to the upper half of the face) rather than error. Two ANOVAs were conducted, one with outliers and one without. Both presented identical main effects and as such the data with the ‘outliers’ were analysed. Greenhouse-Geisser correction was applied.

There was a signficant main effect of ROI, *F* (2.0, 142.5) = 16.03, *p* < .001, η^2^ = .18, with significant dwell time differences between all ROIs, except for between the eyes and non-feature regions (*p* = .061) and the nose and mouth (*p* = .093); there was the most dwell time to the eye region (*M* = 0.35), followed by the non-feature regions (*M* = 0.27), the nose (*M* = 0.22), and the mouth (*M* = 0.15). There was a signficant main effect of stimulus presentation, *F* (1.8, 129.9) = 43.56, *p* < .001, η^2^ = .38, with signficant differences between all types; there was the most dwell time for static EEs (*M* = 0.248), followed by dynamic EEs (*M* = 0.246), and audio-visual EEs (*M* = 0.245).

**Figure 4.**
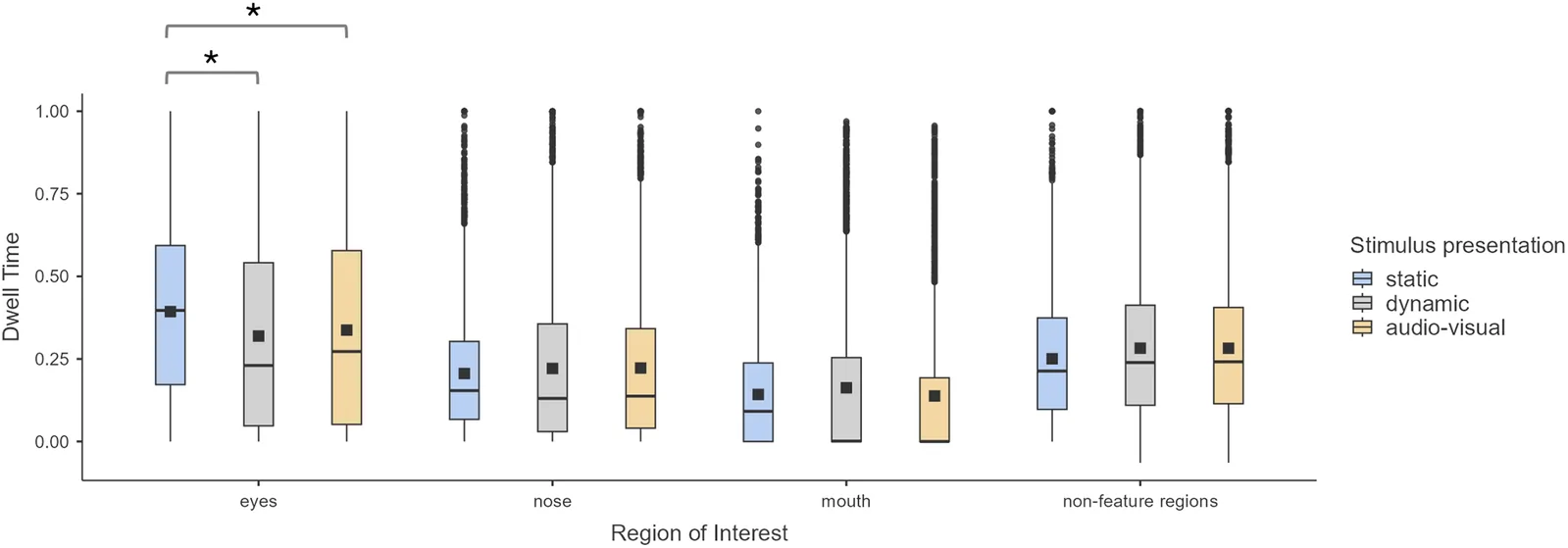
Interaction between pre-defined ROI (eyes, nose, mouth, non-feature regions) and stimulus presentation (static, dynamic, audio-visual) for proportion of dwell time *Note.* ** = *p* < .001, * = *p* < .05. ROI: region-of-interest.

There was a significant interaction between stimulus presentation and ROI, *F* (4.1, 295.3) = 5.84, *p* < .001, η^2^ = .08 (Figure 4). Post hoc tests revealed significantly more dwell time to the eye region compared to the mouth across all stimulus presentations (all *p* < .040). There was signficantly longer dwell time to the eye region for static EEs compared to dynamic (*p* = .004) and audio-visual EEs (*p* = .039), but similar for dynamic and audio-visual EEs (*p* = .899). Dwell time towards the mouth, nose, and non-feature regions was similar across stimulus presentations (*p* > 0.077). Dwell time was the lowest in the mouth region in all conditions.

A three-way ANOVA exploring proportion of dwell time across stimulus presentation (static, dynamic, audio-visual), pre-defined ROI (eyes, nose, mouth, non-feature regions), and emotion expressed (basic six and neutral) is presented in Supplementary materials (S1). There was a significant interaction between ROI and emotion expressed; all emotions showed longer dwell times in the eye region, especially for sadness, fear, and anger, and happiness and disgust showed the highest dwell time for the mouth region. There was a significant interaction between stimulus presentation and emotion expressed; most dwell time differences were non-significant, except for between static and dynamic fear and disgust, and static and audio-visual anger, fear, and disgust. There was also a significant three-way interaction between ROI, stimulus presentation, and emotion expressed. As our main focus is on stimulus presentation, and better understanding static, dynamic, and audio-visual conditions, only the supplementary analysis explored across emotion expressed.

**Research question 2: Are individuals’ eye movement patterns consistent across static, dynamic, and audio-visual EEs?**

**Figure 5.**
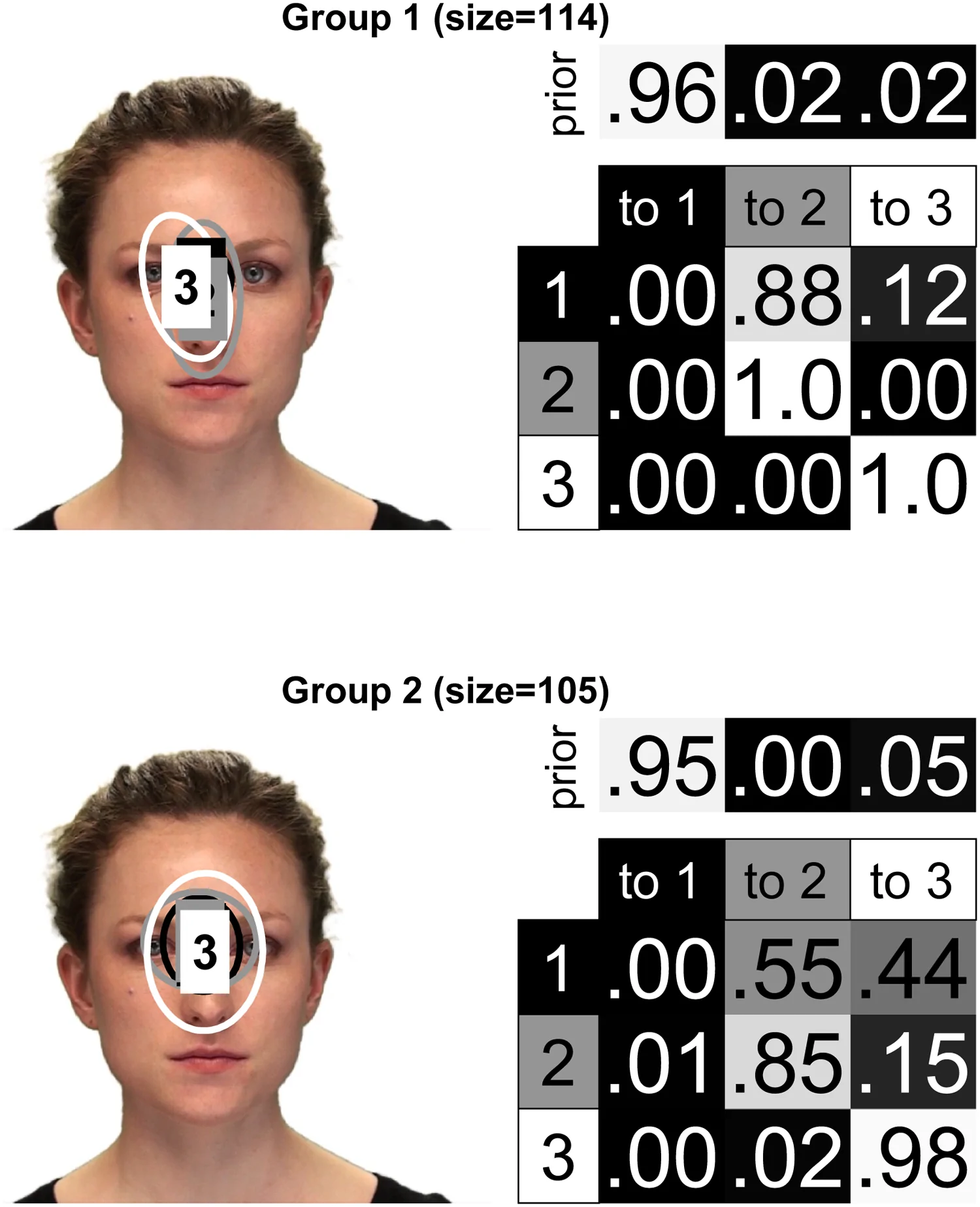
Matrices showing the probability of transitioning from one ROI to another for group 1 (top matrix) and group 2 (bottom matrix). Note. Group 1 starts at ROI 1, goes to ROI 2 (88%), then to ROI 3 (12%). Group 2 starts at ROI 1, then moves to either ROI 2 (55%) or ROI 3 (44%) with similar probability. ROI: region-of-interest.

We used the EMHMM toolbox (Chuk et al., 2014) to generate individual HMMs for each of the 219 datapoints (73 participants x 3 conditions) and separate these into clusters. The analysis revealed two clusters of eye movements. The two clusters in Figure 5 show two different processing patterns. While both cluster groups were more likely to start fixating in a central area of the face focused around the middle of the nose (ROI 1), group 1 (*N* = 114) transitioned to stay in the nose area (ROI 2), staying more centrally focused, whereas group 2 (*N* = 105) transitioned closer to the eye region (ROI 2) or covered a larger area from the lower nose to the mid-forehead (ROI 3) at similar likelihood, showing a more diffused pattern compared to the ROIs for group 1. Thus, group 1 will be referred to as a (centrally) focused pattern, and group 2 will be referred to as a diffused pattern.

The HMMs were significantly different between groups 1 and 2; group 1′s eye movement sequences were significantly more likely to be produced by the centrally focused pattern than the diffused pattern, *t* (113) = 16.08, *p* < .001, *d* = 1.51, and group 2′s sequences were more likely to be produced by the diffused pattern than the centrally focused pattern, *t* (104) = 12.99, *p* < .001, *d* = 1.27. This suggests that the two HMMs are representing two distinct eye movement patterns.

Centrally focused processing patterns were present for 45% of participants in the static condition, 63% in the dynamic condition, and 53% in the audio-visual condition. Looking at the stability of participants’ processing patterns, the majority, 68%, were consistent across static, dynamic, and audio-visual EEs. A small group (8% of participants) was consistent across static and dynamic EEs but switched for audio-visual EEs, another 11% were consistent for dynamic and audio-visual EEs but switched for static EEs, and 12% were consistent for static and audio-visual EEs but switched for dynamic EEs. In short, consistency was the norm. Table 2 presents a more detailed breakdown of the processing patterns which participants employed and the switches between different conditions.

**Table 2.** Count (and percentage) of participants who employed either a focused or diffused processing pattern across static, dynamic, and audio-visual EEs.

		Audio-visual	
	Static pattern: focused (*N* = 33*)*	Focused	Diffused
Dynamic	focused	**27** (**82%**)	*4* (*12%*)
	diffused	2 (6%)	***0***
	**Static pattern: diffused (*N*** **=** **40)**		
Dynamic	focused	***8*** (***20%***)	7 (18%)
	diffused	*2* (*5%*)	**23** (**58%**)

Note. The top half of the table shows the participants who employed a focused processing pattern for static EEs (N = 33), and which patterns they employed across other conditions, and the bottom half of the table shows the participants who employed a diffused processing pattern for static EEs (N = 40), and which patterns they employed across other conditions. The table shows how many participants employed a consistent pattern across all three conditions (**bold**), how many were consistent for static and dynamic EEs but switched for audio-visual EEs (*italics*)*,* how many were consistent for dynamic and audio-visual EEs, but switched for static EEs (***bold italics***), and finally how many were consistent for static and audio-visual EEs, but switched for dynamic EEs (underlined).

EE: emotional expression.

However, it is possible that simple categorisation does not capture more subtle processing pattern shifts. To further explore whether eye movement patterns were consistent across stimulus presentation, we used A-B scale values. Where ‘A’ represents the log-likelihood of the individual's eye movement pattern resembling focused patterns, group 1, and ‘B’ represents the log-likelihood of the pattern resembling diffused patterns, group 2, the A-B scale = (A-B)/(|A| + |B|) (Chan et al., 2018; Chuk et al., 2017b; Zheng & Hsiao, 2023). This measure combines both values to produce an index of similarity encompassing both groups. A more positive value suggests higher similarity to group 1 (focused patterns), and a more negative value suggests higher similarity to group 2 (more diffused patterns) (Zheng & Hsiao, 2023). To understand how people's patterns changed across conditions, we calculated the difference between A-B values for each participant across all three conditions, then converted these into absolute numbers (as for this analysis we were more interested in the amount of change, rather than the directionality of the change). This resulted in three A-B difference values per participant (static–dynamic; static–audio-visual; dynamic–audio-visual), allowing us to quantify the degree of pattern change across conditions within each individual. An A-B difference value of zero would indicate participants’ processing pattern stayed consistent across modalities, but a larger deviation from zero indicates a change in pattern. For ease of interpretation, the A-B difference scores will be discussed in terms of pattern consistency, with values near zero suggesting consistency and values further from zero indicating a lack of consistency.

**Figure 6.**
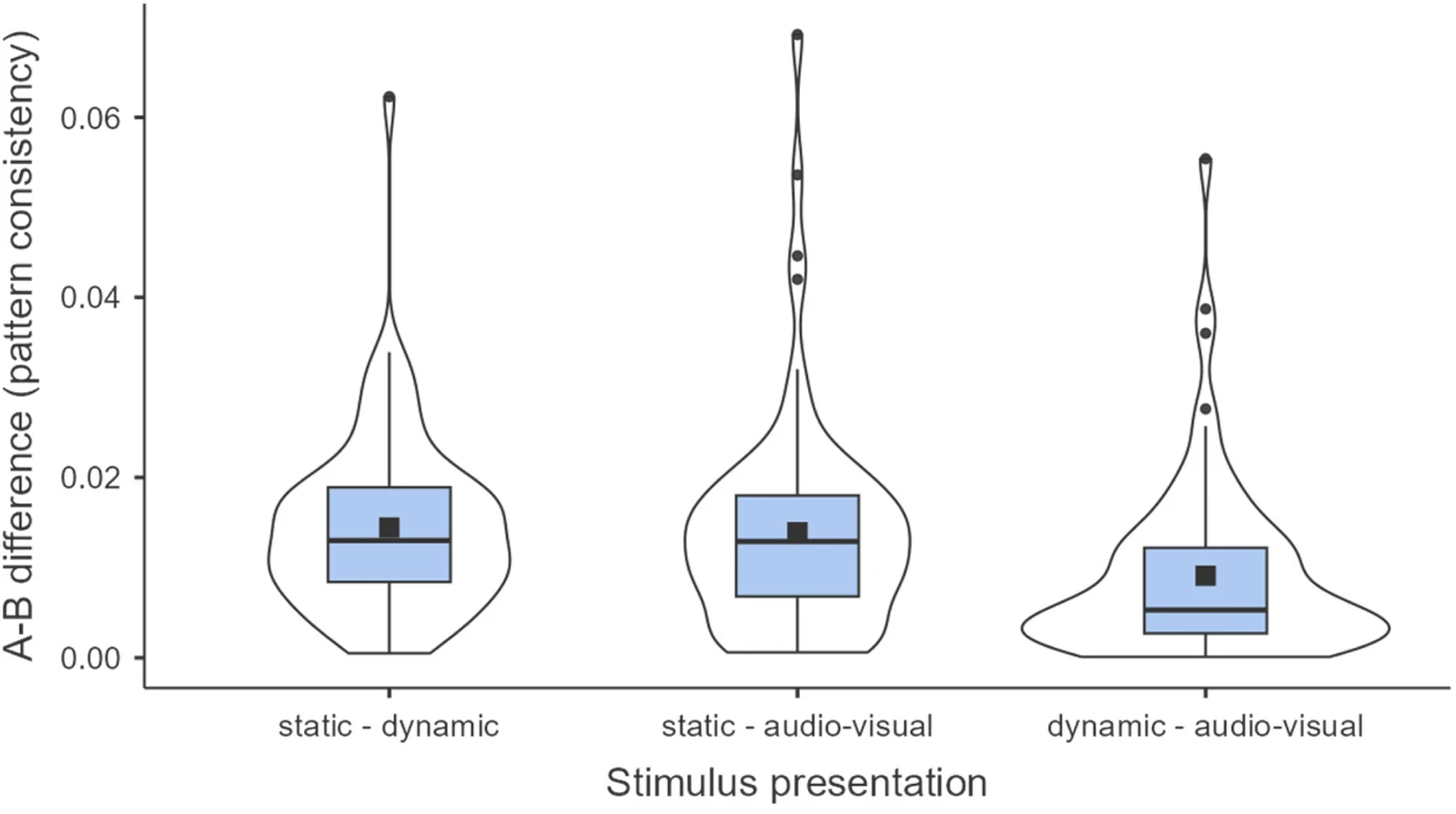
Violin plots depicting processing pattern consistency between static–dynamic conditions (left), static–audio-visual conditions (middle), and dynamic–audio-visual conditions (right). Means close to zero indicate no change between conditions, and scores further away indicate a pattern change. Means depicted by the black box and medians depicted by the black line.

A one-way ANOVA was conducted, with A-B difference (pattern consistency) as the dependent variable and condition comparison (static–dynamic; static–audio-visual; dynamic–audio-visual) as the fixed factor. Normality (explored using Q-Q plot) was violated and outliers were removed (data points from 8 participants were outside of 1.5 times the interquartile range). Sphericity was not violated. Two ANOVAs were conducted, one with outliers and one without, both reported identical main effects and post hoc output. There was a significant difference between pattern consistency across condition comparison, *F* (2, 128) = 17.9, *p* < .001, ηp^2^ = .22. Specifically, individuals changed their processing pattern significantly more between static–dynamic conditions (*M* = 0.013) compared to dynamic–audio-visual conditions (*M* = 0.007), *t* (64) = 5.77, *p* < .001, and between static–audio-visual conditions (*M* = 0.011) compared to dynamic–audio-visual conditions, *t* (64) = 3.67, *p* = .001 (Figure 6). There was no significant difference in changing processing pattern between static–dynamic and static–audio-visual conditions, *t* (64) = 2.04, *p* = .111. While our analysis of stability across categories suggests that eye movement patterns did not change dramatically between static and dynamic or audio-visual images, this analysis suggests that people's eye movement patterns do show subtle shifts when they view static vs moving EEs.

**Research question 3: Are particular processing patterns associated with better emotion recognition accuracy?**

An analysis of whether one processing pattern could impact performance for a particular stimulus presentation was conducted. A two-way ANOVA was conducted with group (1 or 2) and stimulus presentation (static, dynamic, audio-visual) as fixed factors, and average emotion recognition accuracy (proportion correct) as the dependent variable. The interaction between group and stimulus presentation was of interest as this would indicate that a certain pattern employed (focused or diffused pattern) when viewing EEs in a particular presentation condition (static, dynamic, or audio-visual) would impact performance.

**Figure 7.**
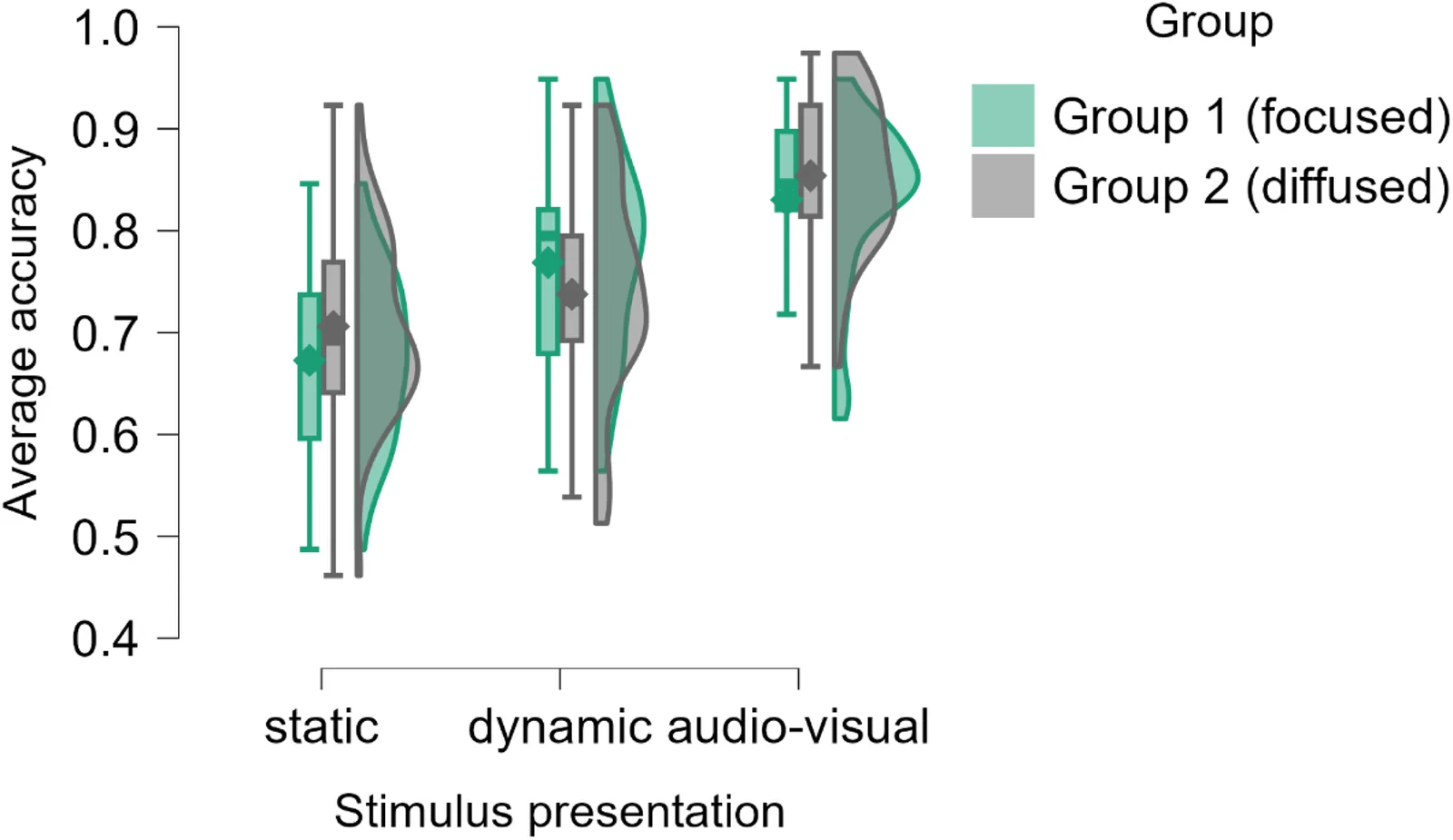
A plot depicting group (1: focused pattern and 2: diffused pattern) and stimulus presentation (static, dynamic, and audio-visual) on average emotion recognition accuracy (proportion correct).

As established in previous papers (Ambadar et al., 2005; Bould & Morris, 2008; Alves, 2013; Blais et al., 2017; Cooper et al., 2024), as number of cues increased, accuracy increased; audio-visual EEs were most accurate (*M* = 0.84), then dynamic (*M* = 0.76), and static EEs had the lowest accuracy (*M* = 0.69). However, there was no significant interaction between group and stimulus presentation, *F* (2, 213) = 2.63, *p* = .075, indicating that processing pattern employed did not impact performance across conditions (Figure 7).

## Discussion

The current study sought to establish eye movement patterns during the emotion processing of static, dynamic, and audio-visual EEs. Given the sparse research using more realistic stimuli, this paper adds relevant and robust findings, derived from two separate analytic approaches, regarding the differences in eye movement patterns for processing static and moving EEs. Our findings show that differences in the visual processing of EEs are due to the inclusion of movement (e.g., static versus moving – dynamic and audio-visual EEs), and that the addition of vocal emotion cues does not substantially affect eye movement patterns, however, vocal cues do help with accurate recognition of EEs.

The first research question focused on whether individuals look in different areas of the face when recognising emotions from static, dynamic, and audio-visual EEs. The findings showed that individuals do focus in different areas, in particular, individuals focused more on the eye region for static EEs compared to dynamic and audio-visual EEs. The difference in eye movements between static and dynamic conditions follows previous adult and child findings (Blais et al., 2017; Prunty et al., 2021). However, there was similar attention to the eye region across dynamic and audio-visual EEs. The observed differences between static and moving conditions could be the result of more effortful scanning of static stimuli to account for the lack of temporal information available. Another key region for recognising EEs is the mouth region (Calvo et al., 2014; Jack & Schyns, 2015; Wegrzyn et al., 2017), and yet similar dwell time was found across conditions. Similar to the explanation above, we would expect a less effortful scanning of the mouth region for audio-visual EEs as a result of the additional vocal information. However, visual attention to the mouth was comparable, suggesting that the eye region may have held more weight than the mouth region for all conditions.

A potential limitation of the moving stimuli used could be the duration; 3–4 seconds may be longer than a natural facial expression, especially for fear and surprise which are known to be quick (Hoffman et al., 2010). However, in order to include linguistic stimuli, which better resemble every day interactions compared to non-linguistic clips (e.g., ‘ah’), longer durations were necessary. It also follows previous dynamic stimuli durations (Prunty et al., 2021). Although, the linguistic element may pose another potential limitation. For the audio-visual stimuli, the facial and vocal EEs were paired with a semantically neutral sentence, which may cause some incongruence between the linguistic content (neutral) and EEs (emotional). The RAVDESS database (Livingstone & Russo, 2018) did intentionally use semantically neutral sentences alongside facial and vocal EEs to ensure performance was driven by emotion cues and not by lexical content. The high accuracy in the audio-visual conditions overall, and not just for neutral expressions, suggests incongruence did not significantly impair participants’ emotion recognition. Audio-visual EEs are optimal to use when exploring emotion recognition due to the inclusion of vocal EEs, which are typically omitted from research despite being a key feature in our everyday interactions.

The second research question focused on whether individuals’ processing patterns were consistent across static, dynamic, and audio-visual EEs. Starting with group categorisation, 68% of participants stayed consistent across conditions. Paparelli et al. (2024) reported similar, with 60% of participants utilising a consistent pattern across both dynamic and static EEs. For those who did change pattern in our study, numbers were comparable between conditions (8–12%). However**,** our main analysis, using A-B scale values, reported that individuals showed more variation in their processing pattern when comparing between static and moving (dynamic and audio-visual) conditions, compared to between dynamic and audio-visual conditions. This difference in findings may suggest that using A-B scale values (and differences between the values across conditions) is more useful to identify subtle variation in processing patterns, which may not be reflected in group categorisation changes. Zheng and Hsiao (2023) also used a similar method of exploring the change in A-B scale between their audio-visual and video-only conditions for accuracy. Thus, future research should incorporate more subtle measures of pattern consistency (e.g., A-B scale difference).

The third research question focused on whether a particular processing pattern was associated with higher accuracy; is there an optimal pattern to enhance performance? The data showed performance was comparable across patterns, suggesting neither one showed an accuracy advantage. These results mirror Paparelli et al.'s (2024) findings, but they contrast with work using HMMs to study eye movement patterns during face identification tasks, which has consistently reported an advantage for more analytic eye movement patterns (e.g., Chan et al., 2018; Chuk et al., 2017a; Chuk et al., 2017b). Although one pattern prevailing the other would have been helpful to create interventions to improve emotion recognition abilities, it is useful to know that one group (e.g., centrally focused processers) are no more disadvantaged than others when processing EEs. This may be explained by the fact that individual's preferred processing pattern has been found to be the most effective for accurate recognition (Peterson & Eckstein, 2013; Yitzhak et al., 2022). The findings suggest that eye movements are not driving individuals’ emotion recognition accuracy. It would be interesting for future research to explore where the differences do stem from, e.g., is it another process involved in emotion recognition, before labelling, which is impacting the accurate recognition/labelling of emotions? For example, it could be linked to individuals’ preferred location for their first saccade in the early stages of emotion recognition (Peterson & Eckstein, 2013). This could not be explored in the current study as it requires a different experimental set up (e.g., jittering of the initial fixation and/or stimulus location, which was not implemented in the current study). Thus, future research could explore whether these preferred early fixations are consistent across tasks and conditions, as well as using younger samples to explore when these patterns emerge and their stability over development. Alternatively, differences in performance may reflect later-level cognitive processes associated with linking perceptual inputs to complex symbolic and semantic information (Adolphs, 2002). Future work might consider employing a variety of different emotion-related tasks (e.g., matching, matching-to-label, free-viewing and free-labelling) to determine the contribution of perceptual processes (e.g., individual variation in eye movement patterns) and cognitive mechanisms to performance in naturalistic emotion recognition tasks.

Bringing all the findings together, the main consistent conclusion is that static EEs are processed somewhat differently to dynamic and audio-visual EEs, whereas dynamic and audio-visual follow broadly similar patterns. Thus, in most of our analyses, variation in eye movements associated with emotion processing was related to movement (e.g., static versus dynamic and audio-visual EEs), rather than based on the number of cues available (e.g., static or dynamic facial expression versus audio-visual EEs). This is expected as static and moving EEs present different information. Static images present spatial information of an EE, whereas dynamic videos present both spatial and temporal information, including the direction, quality, and speed of movement (see Krumhuber et al., 2023 for an in-depth review). As such, we expect different eye movement patterns due to the inclusion of movement. However, it is still important to explore static versus dynamic conditions to better understand exactly where, and how great, the processing differences are, as well as what conclusions can be drawn depending on stimulus presentation. On the contrary, both the dynamic and audio-visual conditions involve motion and have the same amount of visual information available, so similar eye movement patterns would be expected.

Regarding accuracy, auditory information processing must be contributing to more accurate performance during audio-visual presentation, but this would not be visible in eye movement patterns (although it was apparent in our analysis). Thus, to take this further we need a multimodal study which incorporates eye tracking alongside neuroimaging methods, e.g., EEG or fMRI. As previous research tends to omit audio-visual EEs, it was unclear how they were processed in relation to static and dynamic EEs. Zheng and Hsiao (2023) did include audio-visual EEs (but did not include static) and reported that eye movement patterns were similar across dynamic and audio-visual EEs, supporting our findings. The main implication of static EEs being processed differently to moving EEs is that it allows us to carefully consider previous research which has typically used static stimuli. This may suggest that previous research employing static EEs are useful in providing information on how we process and recognise static EEs, but the findings may struggle to generalise and apply to moving EEs, and more importantly, to real-world interactions. Thus, this highlights the need for future research to use more realistic and ecologically valid moving stimuli when exploring emotion recognition.

## Conclusion

The current study has addressed existing gaps in the literature regarding stimulus presentation (static, dynamic, and audio-visual EEs), whether individuals’ eye movement patterns differ across these conditions, and how this is related to emotion recognition accuracy. The findings showed that individuals focused on different areas of the face when processing static, dynamic, and audio-visual EEs (static EEs were more eye focused than dynamic and audio-visual EEs), and that individuals’ processing patterns were largely consistent – although most likely to change between static and moving EEs (dynamic and audio-visual), however, a particular processing pattern was not linked to improved accuracy performance. As eye movements are not the driving force behind poor emotion recognition abilities, future research should focus on where the difficulties do lie. Ultimately, it seems that EEs are processed differently dependant on movement rather than the amount of emotion cues available (e.g., facial-only versus audio-visual EE). This highlights important considerations for previous research which used static EEs and urges future research to use more realistic moving stimuli to be able to better generalisable to real-world social interactions.

## Supplemental Material

sj-docx-1-pec-10.1177_03010066261439924 - Supplemental material for Consistency and changes in eye movements during static, dynamic, and audio-visual emotion processing Supplemental material, sj-docx-1-pec-10.1177_03010066261439924 for Consistency and changes in eye movements during static, dynamic, and audio-visual emotion processing by Holly Cooper, Ben J. Jennings, Veena Kumari, Caroline Di Bernardi Luft and Rachel J. Bennetts in Perception

## Notes

1. The same data are also presented in Zheng and Hsiao (2020) for conference proceedings, but here the paper will be referred to.
