# Supplementary material for: Consistency and changes in eye movements during static, dynamic, and audio-visual emotion processing
Source: Perception. 2026 Apr 29;55(10):938–56. doi: 10.1177/03010066261439924 (PMC13578650; doi:10.1177/03010066261439924)
Supplement: Supplementary file 1 [file sj-docx-1-pec-10.1177_03010066261439924.docx]

**Supplementary material for ‘Consistency and changes in eye movements during static, dynamic, and audio-visual emotion processing’**

Holly Cooper, Ben J. Jennings, Veena Kumari, Caroline Di Bernardi Luft, and Rachel J. Bennetts

**S1**

A three-way ANOVA was employed to examine the main effects of stimulus presentation (static, dynamic, audio-visual), pre-defined ROI (eyes, nose, mouth, non-feature regions), and emotion expressed (basic six and neutral) on proportion of dwell time. Normality was checked with Q-Q plots and presented as slightly non-normal. Outliers were checked, but as with the main analyses, it seemed most lied within dwell time to the mouth region across conditions. Two ANOVAs were conducted, one with outliers and one without. The main effects and interactions differed: the ANOVA with outliers reported significant effects and interactions, whereas the ANOVA without outliers reported no significant interaction between stimulus presentation and emotion expressed and between stimulus presentation and region of interest. The ANOVA without outliers showed singularity issues, due to the extent of missing values within the mouth region of interest. Thus, the data with outliers were analysed, but is to be interpreted cautiously. Sphericity was violated and Greenhouse-Geisser correction applied.

There was a significant main effect of ROI, *F* (2.0, 141.5) = 16.38, *p* < .001, η^2^ = .19, with significant differences in dwell time between all regions, except for between the eyes and non-feature regions (*p* = .077) and between the nose and mouth (*p* = .071). There was a significant main effect of stimulus presentation, *F* (1.7, 121.0) = 42.15, *p* < .001, η^2^ = .37, with significant differences in dwell time between all types (all *p* <.001). There was a significant effect of emotion expressed, *F* (4.7, 339.0) = 17.14, *p* < .001, η^2^ = .19, with significant differences between all emotions, except between happy and anger (*p* = .828), happy and fear (*p* = 1.00), happy and disgust (*p* = .997), sad and surprise (*p* = .677), sad and neutral (*p* = 1.00), anger and fear (*p* = .934), anger and disgust (*p* = .989), fear and disgust (*p* = .995), and surprise and neutral (*p* = .434).

There was a significant interaction between ROI and stimulus presentation, *F* (4.2, 299.6) = 5.55, *p* < .001, η^2^ = .07, which is already discussed in the main paper for the two-way ANOVA without emotion expressed. There was a significant interaction between ROI and emotion expressed, *F* (10.5, 756.9) = 15.06, *p* < .001, η^2^ = .17; all emotions showed longer dwell times in the eye region, especially for sadness, fear, and anger, and happiness and disgust showed the highest dwell time for the mouth region (see Figure 1). There was also a significant interaction between stimulus presentation and emotion expressed, *F* (6.5, 465.2) = 5.05, *p* < .001, η^2^ = .07; most dwell time differences were non-significant, except for between static and dynamic fear and disgust, and static and audio-visual anger, fear, and disgust (see Figure 2). There was a significant three-way interaction between the fixed factors, *F* (19.8, 1428.1) = 5.00, *p* < .001, η^2^ = .07. As with most three-way interactions, this is to be interpreted with caution. Full post hoc comparison tables for the two-way interactions and the three-way interaction are available upon request. They were not included in supplementary as the tables span ~20 and 73 pages, respectively.

**Figure 1**

*Interaction between pre-defined ROI (eyes, nose, mouth, non-feature regions) and emotion expressed (basic six emotions and neutral) for proportion of dwell time*

**Figure 2**

*Interaction between stimulus presentation (static, dynamic, audio-visual) and emotion expressed (basic six emotions and neutral) for proportion of dwell time.*
